# The Role of Ruminants as Sentinel Animals in the Circulation of the West Nile Virus in Tunisia

**DOI:** 10.3390/pathogens14030267

**Published:** 2025-03-08

**Authors:** Ahmed Ouni, Hajer Aounallah, Wafa Kammoun Rebai, Francisco Llorente, Walid Chendoul, Walid Hammami, Adel Rhim, Miguel Ángel Jiménez-Clavero, Elisa Pérez-Ramírez, Ali Bouattour, Youmna M’Ghirbi

**Affiliations:** 1 Laboratoire des Virus, Vecteurs et Hôtes (LR20IPT02), Institut Pasteur de Tunis, Université Tunis El Manar, Tunis 1002, Tunisia; ahmed.ouni@fst.utm.tn (A.O.); aounallahhajer@gmail.com (H.A.); walid.hammami@pasteur.tn (W.H.); adel.rhaim@pasteur.tn (A.R.); ali.bouattour@pasteur.tn (A.B.); 2 Medical Research Direction, Ministry of Health, Tunis 1006, Tunisia; kammoun.wafa@gmail.com; 3 Centro de Investigación en Sanidad Animal (CISA-INIA), CSIC, Valdeolmos, 28130 Madrid, Spain; dgracia@inia.csic.es (F.L.); majimenez@inia.csic.es (M.Á.J.-C.); elisaperezramirez@gmail.com (E.P.-R.); 4 Circonscription of Animal Production of Ben Guerdane, Médenine 4160, Tunisia; walid.chandoul80@gmail.com; 5 CIBER of Epidemiology and Public Health (CIBERESP), 28029 Madrid, Spain

**Keywords:** West Nile Virus, serosurvey, cELISA, VNT, RT-qPCR, ruminants, southern Tunisia

## Abstract

Outbreaks of the West Nile Virus (WNV) have increased significantly in recent years in the Mediterranean region, including Tunisia. To understand the risks for animal and human health and to mitigate the impact of future outbreaks, comprehensive viral surveillance in vertebrate hosts and vectors is needed. We conducted the first serosurvey for the WNV in ruminants in southern Tunisia using the ELISA test and confirmed it with the micro-virus neutralization test (VNT). Antibodies were detected by the ELISA test in camels (38/112), sheep (9/155), and goats (7/58), and six samples were doubtful (five camels and one sheep). The ELISA positive and doubtful sera (*n* = 60) were further analyzed to confirm the presence of specific anti-WNV and anti-Usutu virus (USUV) antibodies using the micro-virus neutralization test (VNT). Out of the 60 sera, 33 were confirmed for specific WNV antibodies, with an overall seroprevalence of 10.15% [95% CI: 7.09–13.96]. The high seroprevalence observed in camels (22.3%) suggests their potential use as sentinel animals for WNV surveillance in southern Tunisia. The viral genome, and consequently active circulation, could not be detected by real-time RT-qPCR in blood samples. Ongoing surveillance of the WNV in animals, including camels, sheep, and goats, may be used for the early detection of viral circulation and for a rapid response to mitigate potential outbreaks in horses and humans.

## 1. Introduction

Bordered by Libya and Algeria, Tunisia is open to the sub-Saharan region of Africa, a prime area for the emergence of the arbovirus. Several viruses transmitted by arthropods have been reported in livestock in Tunisia [1,2]. Arboviruses, especially those transmitted by mosquitoes, pose a significant threat for human and animal health and require rigorous surveillance and control strategies. Among them, the West Nile Virus (WNV) is a zoonotic arbovirus of particular concern in Tunisia, since it has been reported in humans [3], animals, and mosquitoes [2,4,5,6].

The WNV is a single-stranded RNA virus of the *Flaviviridae* family and the genus *Orthoflavivirus* transmitted through an enzootic cycle involving birds and mosquitoes, with occasional spillover to humans and horses [7]. First identified in the West Nile region of Uganda in 1937 [8], the WNV has since spread globally, and is now present on all continents except for Antarctica [9,10]. Birds act as amplifying hosts, and their migratory patterns have significantly influenced the re-emergence and global spread of the virus [11]. The WNV infects ornithophilic mosquitoes when they feed on the blood of infected birds and transmit the virus to the next susceptible host [12].

In horses and humans, WNV infections cause a range of symptoms from mild flu-like symptoms to severe neurological disorders, including meningitis, encephalitis, and acute flaccid paralysis [13,14]. However, both species are considered dead-end hosts because they do not produce sufficient viremia levels to infect mosquitoes; therefore, they do not contribute to the transmission cycle [15].

During this decade, the WNV has circulated in North Africa, including in Tunisia [16,17,18,19,20]. This reemergence in the region is attributed to the presence of bird reservoirs and favorable environmental conditions, especially climatic, for the abundance of *Culex* mosquito species, mainly *Cx. pipiens* and *Cx. perexiguus*, which are the main vectors of the WNV in Tunisia [2,21,22,23,24]. WNV outbreaks occurred in Tunisia in approximately six-year cycles in 1997, 2003, 2012, 2018, and 2023 [3,25,26,27,28,29]. The first evidence of WNV circulation in horses occurred in 2014 in the oases of Tozeur, in southern Tunisia [5]. Other studies have reported antibodies against the WNV in dromedaries, birds and equids, indicating exposure to the virus [6,30,31,32,33]. Understanding the epidemiology and prevalence of the WNV in Tunisia is crucial for implementing effective control and prevention strategies. Ruminants, including sheep, goats, and camels, could play a role as sentinel species. Thus, the aims of this study were (1) to determine the WNV seroprevalence rates in these species, using the ELISA test for a first screening and a confirmation by VNT and (2) to evaluate active circulation of the virus using molecular detection techniques on the blood samples from these animals.

## 2. Materials and Methods

### 2.1. Study Area and Sampling

This study was conducted between 2022 and 2023 in four localities in southern Tunisia in the two governorates of Tozeur and Medenine. In Tozeur, three sampling localities close to the Algerian border were investigated: Hezoua [33°44′–33°48′ N and 7°34′–7°35′ E], Tamaghza [34°26′–34°27′ N and 7°58′–8°02′ E], and Nafta [33°51′–33°52′ N and 7°52′–7°53′ E]. In Medenine we investigated the locality of Ben Guerden [33°10′–33°00′ N and 11°05′–11°23′ E], bordering Libya. These arid and Saharan regions typically experience extremely high temperatures, low relative humidity, and minimal annual precipitation of less than 100 mm.

Serum samples were collected from traditional farms, from randomly selected animals, as per the recommendations of the Tunisian State Veterinary Office, to ensure that the samples were representative of the local traditional management system. Sex, age, breed, and general health status of each animal were recorded. During the blood sampling, meticulous attention was paid to the animals’ welfare. The veterinarian exercised utmost care to ensure that the samples were taken from the jugular vein in an aseptic manner. For RNA detection, only one milliliter of whole blood was collected and placed in tubes containing EDTA, as anticoagulant, and 2 mL of DNA RNA Shield (Zymo Research, Irvine, CA, USA). In addition, five milliliters of peripheral blood were collected in tubes without a coagulant for serological analyses. Sera were processed by centrifugation, abiding by all necessary biosafety precautions, and stored at −20 °C until testing.

### 2.2. Serological Investigation of WNV Infection in Ruminants

#### 2.2.1. ELISA Competition Test

Serum samples from camels, sheep, and goats were analyzed using a commercial ELISA kit that detects WNV anti-E protein antibodies (ID Screen West Nile Competition Multi-species ID-vet Innovative Diagnostics, Montpellier, France). The cELISA protocol was followed as per the manufacturer’s instructions. The OD for each sample was read at a wavelength of 450 nm. Signal-to-noise percentage (S/N%) was computed as follows: S/N% = (OD of each sample/OD of negative control) × 100. A sample exhibiting an S/N% value less than or equal to 40% was classified as positive; a value exceeding 50% was considered negative. Any S/N% value ranging from greater than 40% to less than 50% was designated as doubtful.

#### 2.2.2. Virus-Neutralization Test (VNT)

The sera considered positive or doubtful in the cELISA assay were subjected to a confirmatory micro-virus neutralization test (VNT), performed against the WNV strain E101 and USUV SAAR-1776 (Genbank accession numbers AF260968 and AY453412) to confirm the presence of specific neutralizing antibodies against the WNV and differentiate from the antibodies produced against USUV, as both viruses can cross-react in the cELISA.

The VNT was performed in 96-well microtiter plates, as previously described [34], based on the method validated by the World Organisation for Animal Health manual (WOAH) [35]. Neutralizing the immune response was assigned as specific for either WNV or USUV when the VNT titer was at least four times higher than the titer obtained for the other virus [36]. When the titer differences were below this threshold, the result was considered inconclusive and assigned as an undetermined flavivirus.

### 2.3. Molecular Analysis

#### 2.3.1. RNA Extraction

RNA extraction was performed on 140 µL of blood using the QIAamp Viral RNA Mini Kit (Qiagen, Hilden, Germany) as per the manufacturer’s instructions. The final elution volume of the extracted viral RNA was 60 µL. The quantity and the quality of the extracted RNA were measured using a NanoDrop spectrophotometer (NanoDrop 1000c; Thermo Scientific, Wilmington, DE, USA)).

#### 2.3.2. RT_qPCR Test

We used specific primers WN10533-10552 (AAG TTG AGT AGA CGG TGC TG) and WN10625-10606 (AGA CGG TTC TGA GGG CTT AC) to amplify a conserved 92 bp region spanning nucleotides from 10,533 to 10,625 of the WNV 3′-noncoding region. For detection we used the probe WN10560-10579 (CTC AAC CCC AGG AGG ACT GG) [37], labeled with 6-carboxyfluorescein (FAM) at its 5′-end and a TAMRA-fluorescent reagent at its 3′-end. RT-qPCR was performed in a 20 µL reaction using the AgPath-IDTM One-step RT-PCR Kit (Thermo Fisher Scientific, Austin, TX, USA). RNA eluate (5 µL from each sample) was used for amplification. The thermal cycling conditions consisted of 10 min at 45 °C, 10 min at 95 °C, followed by 45 cycles of 15 s at 95 °C and 45 s at 60 °C. The assays were performed in duplicate, and samples with threshold cycle (Ct) values under 40 were considered positive. In each run, the RNA obtained from the Tunisian outbreak in 1997 (PaH001, GenBank Genome Accession: AY268133) was used as the positive control, while pure water was used as the negative control.

### 2.4. Statistical Analysis

Statistical analyses were performed using GraphPad Prism version 8.4.3 (GraphPad Software, Inc., La Jolla, CA, USA) to determine the seroprevalence of the WNV with 95% confidence intervals (CI). Differences in seroprevalence rates between animal species and localities were evaluated using the one-way analysis of variance (ANOVA). A Chi-square test was performed to compare the WNV seroprevalence rates between the animal’s age and sex groups. Statistical significance was set at a two-tailed *p*-value < 0.05.

## 3. Results

A total of 112 local breed camels (*Camelus dromedarius*) were tested (16 males and 96 females), aged between 9 months and 18 years (average age of 9.77 ± 4.03 years). In addition, 155 local breed sheep were tested (15 males and 140 females), with ages ranging from 6 months to 9 years (average of 4.46 ± 2.32 years). We also sampled 58 local breed goats (4 males and 54 females), aged between 8 months and 10 years (average of 4.45 ± 2.20 years).

### 3.1. WNV Seroprevalence in Ruminants

Using cELISA, the overall seroprevalence of flavivirus antibodies was 16.61% among the three tested animal species (54/325; 95% CI: 12.74–21.12). After the VNT, 10.15% [33/325; 95% CI: 7.09–13.96] were confirmed positive for specific WNV antibodies (Table 1).

The VNT showed that 22.32% [25/112; 95% CI: 15.00–31.16], 5.2% [3/58; 95% CI: 1.08–14.38] and 3.2% [5/155; 95% CI: 1.06–7.37] of camels, goats and sheep, respectively, were positive for WNV antibodies (Table 1). The difference in WNV seropositivity rates between species was significant (*p* < 0.05; Table 1). In addition, the seropositivity rates in camels differed significantly between the localities (*p* < 0.001). Specific WNV antibodies in goats were detected only in Hezoua 9.7% [3/31; 95% CI: 2.04–25.75] with no significant differences between localities (*p* = 0.054). Similarly, only sheep from Hezoua showed specific WNV antibodies with a seroprevalence rate of 7.8%; [5/64; 95% CI: 2.58–17.30] (Table 1).

Camels exhibited a significant age-dependent WNV seroprevalence (young: 3.57% [4/112; 95% CI: 0.98–8.89] compared to adults: 18.75% [21/112; 95% CI: 12.0–27.22], *p* < 0.001). In contrast, sheep (*p* = 0.22) and goats (*p* = 0.62) showed no age-associated differences (Table 1). Camels exhibited a significant difference in WNV seroprevalence between sexes. Specifically, females had a higher seroprevalence 20.54% [23/112; 95% CI: 13.49–29.20] than males 1.79% [2/112; 95% CI: 0.98–8.89], *p* < 0.001. In contrast, neither goats (*p* > 0.05) nor sheep (*p* > 0.05) showed significant sex-associated differences in seroprevalence (Table 1).

### 3.2. Molecular Investigation

All blood samples (*n* = 325) were negative for the WNV RNA by real-time RT-qPCR.

## 4. Discussion

West Nile fever is a complex disease with a multifaceted epidemiology that involves various animal species and arthropod vectors. Understanding the role of these species in the WNV transmission cycle is crucial for effective monitoring and control. In this study, we investigated the role of camels and small ruminants (sheep, goats) in WNV surveillance in southern Tunisia. The cELISA revealed an overall flavivirus seroprevalence of 16.6% in 325 animals, of which 10.15% had WNV-specific neutralizing antibodies as confirmed by the VNT. Notably, camels exhibited the highest seroprevalence (22.32%), followed by goats (5.2%) and sheep (3.2%). Similar results were reported in Egypt in a locality sharing the same bioclimatic conditions as southern Tunisia. Using the cELISA test they detected seroprevalence rates of 40% in camels, 3.5% in sheep, and 5.3% in goats [38]. Megenas et al. [39] reported a WNV seroprevalence of 20.7% in camels, 11.6% in goats, and 3.5% in sheep in the Afar region of northeast Ethiopia. These findings are consistent with our results, which confirm the utility of camels in WNV surveillance in arid zones.

Camels are of special interest in the study of WNV circulation due to their extensive presence across Saharan arid and semi-arid regions. A serological survey in Morocco showed an increase in WNV seroprevalence in camels from 10% in 2003 to 13% in 2009, suggesting frequent exposure to infected mosquito populations [40]. Camels exhibit a higher seroprevalence of the WNV compared to small ruminants, which is likely due to a longer lifespan, which increases their cumulative exposure to mosquito bites and thus elevates seropositivity rates. This hypothesis is supported by the observed age-dependent increase in WNV seroprevalence among camels, favoring adults. Notably, female camels showed significantly higher anti-WNV antibody levels than males, as also observed by Baba et al. [41]. Furthermore, the low seroprevalence of the WNV in sheep and goats is noteworthy and differs from previous studies [42]. This discrepancy may be attributed to various factors, such as differences in sampling timing, variations in WNV circulation dynamics, or the presence of competent mosquito vectors in different regions [43,44].

Previous clinical and serological studies have confirmed the circulation of the WNV in Tunisia, with documented cases in humans, equines, and birds, particularly across the northeastern governorates, the eastern coast, and the southern lowlands [5,23]. In the southern studied localities (arid to Saharan), WNV circulation is maintained within oasis ecosystems, which are suitable habitats for birds and are critical for the survival of mosquito populations that transmit the virus [31]. Additionally, their proximity to Chott el Djerid, a major salt lake and migratory birds’ refuge, increases the risk of virus introduction and dispersion. Virological surveillance in these areas is crucial for understanding WNV dynamics and potential health risks. Indeed, in the oasis of Tozeur, Bargaoui et al. [31] reported a seroprevalence of 52% in the equine samples tested by the cELISA test. In the oasis of Kébili (bordering Tozeur), Ben Hassine et al. [5] reported a seroprevalence of 58.2% in 284 tested equids. Furthermore, in the oasis of Tozeur, where an outbreak of the WNV fever affected several citizens in 2012 [45], WNV antibodies were detected in wild birds [46,47]. These results underscore the need to continue investigating WNV circulation in these localities, with a particular focus on sentinel species that could contribute to monitoring the circulation of the virus in this ecosystem.

Whereas ruminants are considered dead-end hosts for the WNV, they can play an important role as epidemiological sentinels [17,48,49]. Even though they do not amplify the virus, their seropositivity offers valuable insights into viral circulation as it reflects the intensity of transmission cycles involving mosquitoes and birds [50]. An effective surveillance system requires a coordinated framework that synchronizes the monitoring of vector populations, environmental conditions, and host serological responses using sentinel species, such as camels and other ruminants, as proposed in this work. Future studies should adopt a multidisciplinary approach to enhance our understanding of WNV transmission dynamics and promote a rapid outbreak response through a collaboration among the veterinary, ecological, and public health sectors.

In Tunisia, the WNV has been detected in humans and *Culex* mosquitoes [2,3], while in our study, no viral RNA was detected in 325 blood samples from camels, goats and sheep, tested by RT-qPCR. Even so, 10.15% of these animals exhibited WNV antibodies. The absence of viral RNA in ruminants does not exclude the circulation of the WNV given the low likelihood of detecting the viral genome in dead-end hosts through active surveillance [9], due to the short viremia elicited by infection in these hosts. Indeed, viral RNA in the blood of dead-end hosts often lasts a few days [14], which poses a challenge for RNA detection when sampling does not coincide with peak viremia. Consequently, integrating serological assays with molecular techniques is critical for a comprehensive assessment of WNV circulation [2]. A recent study in Tunisia also detected other arboviruses, including the flavivirus Usutu virus (USUV) and the alphavirus Sindbis virus (SINV), in mosquitoes and horses [2]. Despite the paucity of comprehensive prevalence data for these viruses, their presence suggested a possible serological cross-reactivity, which may account for the observed discrepancies between the cELISA and VNT results. Consequently, despite cELISA’s sensitivity in initial screenings, its cross-reactivity with other flaviviruses, especially in the areas where multiple flaviviruses co-circulate [34], necessitated the use of confirmatory assays such as the VNT to ensure diagnostic specificity and accuracy in WNV serological surveillance [51,52]. This two-step approach aligns with the WOAH guidelines recommending VNT as the gold standard for serological confirmation [35]. Studies from diverse epidemiological settings including Bulgaria, the Netherlands, and Malaysia, have reported significant discrepancies between the cELISA and VNT results, reinforcing the importance of confirmatory testing [53,54,55].

## 5. Conclusions

In conclusion, our study confirmed the circulation of the WNV in Tunisia’s oasis regions and reinforced the previous findings that linked the WNV to neurological diseases in humans. Furthermore, the data highlighted the critical role of camels and small ruminants as sentinel species for effective WNV surveillance in endemic areas, thereby enhancing early detection and control efforts.

## Figures and Tables

**Table 1:** Seroprevalence of WNV in camels, sheep, and goats in the prospected provinces.

Animal Species	Localites	Tested Serum Samples	cELISA Seropositive/Nb of Tested Animals (%) [95% CI]	Specific WNV Antibodies by VNT/Nb of Tested Animals (%) [95% CI]	Age Groups	Specific WNV Antibodies by VNT/Nb of Tested Animals (%) [95% CI]	Sex	Specific WNV Antibodies by VNT/Nb of Tested Animals (%) [95% CI]
**Camel**	Ben Guerden	32	1/32 (3.1) [0.08–16.22]	1/32 (3.1) [0.08–16.22]	1–5 years Young	4/112 (3.57) [0.98–8.89]	Females	23/112 (20.54) [13.49–29.2]
	Hezoua	58	29/58 (50) [36.58–63.42]	20/58 (34.5) [22.49–48.12]				
	Nafta	20	7/20 (35) [15.39–59.22]	4/20 (20) [5.73–43.66]	>5 years Adults	21/112 (18.75) [12.0–27.22]	Males	2/112 (1.79) [0.98–8.89]
	Tamaghza	2	1/2 (50) [1.26–98.74]	0/2 (0) [0–84.19 *]				
**Total**		122	39/112 (34.8) [26.07–44.4]	25/112 (22.32) [15.00–31.16]				
***p*** **-value**				<0.001	<0.001		<0.001	
**Goat**	Ben Guerden	10	0/10 (0) [0.0–30.85 *]	0/10 (0) [0–30.85 *]	<1 year Kids	2/58 (3.45) [0.42–11.91]	Females	3/58 (5.2%) [1.08–14.38]
	Hezoua	31	6/31 (19.4) [7.45–37.47]	3/31 (9.7) [2.04–25.75]				
	Tamaghza	17	0/7 (0) [0–40.96 *]	0/17 (0) [0–19.51 *]	>1 year Adults	1/58 (1.72) [0.04–9.24]	Males	0/58 (0%) [0–6.16*]
**Total**		58	6/58 (10.3) [3.89–21.17]	3/58 (5.2%) [1.08–14.38]				
***p*** **-value**				0.054	0.62		0.242	
**Sheep**	Ben Guerden	52	0/52 (0) [0–6.85 *]	0/52 (0) [0–6.85 *]	<1 year Lamb	1/155 (0.65) [0.02–3.54]	Females	4/155 (2.58) [0.71–6.48]
	Hezoua	64	9/64 (14.1) [6.64–25.02]	5/64 (7.8) [2.58–17.30]				
	Tamaghza	39	1/39 (2.6) [0.06–13.48]	0/39 (0) [0–9.03 *]	>1 year Adults	4/155 (2.58) [0.71–6.48]	Males	1/155 (0.65) [0.02–3.54]
**Total**		155	10/155 (6.5) [3.14–11.54]	5/155 (3.2%) [1.06–7.37]				
***p*** **-value**				0.005	0.22		0.367	
**Total**		325	54/325 (16.6) [12.74–21.12]	33/325 (10.15) [7.09–13.96]				
***p*** **-value**				0.016				

*: One-sided 97.5% confidence interval.

## Data Availability

The datasets supporting the conclusions of this article are included within the article.

## Abbreviations

The following abbreviations are used in this manuscript:

cELISA	competition Enzyme-Linked Immunosorbent Assay
SINV	Sindbis Virus
USUV	Usutu Virus
VNT	Virus Neutralization Test
WNV	West Nile Virus
